# Imaging the Response to DNA Damage in Heterochromatin Domains

**DOI:** 10.3389/fcell.2022.920267

**Published:** 2022-06-02

**Authors:** Audrey Chansard, Enrico Pobega, Pierre Caron, Sophie E. Polo

**Affiliations:** Epigenetics and Cell Fate Centre, UMR7216 CNRS, Université Paris Cité, Paris, France

**Keywords:** confocal microscopy, DNA damage, DNA repair, heterochromatin, laser micro-irradiation, UV

## Abstract

The eukaryotic genome is assembled in a nucleoprotein complex called chromatin, whose organization markedly influences the repair of DNA lesions. For instance, compact chromatin states, broadly categorized as heterochromatin, present a challenging environment for DNA damage repair. Through transcriptional silencing, heterochromatin also plays a vital role in the maintenance of genomic integrity and cellular homeostasis. It is thus of critical importance to decipher whether and how heterochromatin affects the DNA damage response (DDR) to understand how this chromatin state is preserved after DNA damage. Here, we present two laser micro-irradiation-based methods for imaging the DDR in heterochromatin domains in mammalian cells. These methods allow DNA damage targeting to specific subnuclear compartments, direct visualization of the DDR and image-based quantification of the repair response. We apply them to study DNA double-strand break repair pathways in facultative heterochromatin and the repair of UV photoproducts in constitutive heterochromatin. We discuss the advantages and limitations of these methods compared to other targeted approaches for DNA damage induction.

## 1 Introduction

The induction of DNA damage challenges genome integrity and cellular homeostasis (Jackson and Bartek, 2009). To counteract this, a plethora of DNA repair pathways operate to resolve the broad range of different DNA lesions that the cells encounter (Hoeijmakers, 2009). DNA repair processes can vary according to the cell cycle or to the transcriptional activity of the damaged locus. In addition, DNA repair in eukaryotic cells occurs in a chromatin context, which also exerts a strong influence on the repair response (Ferrand et al., 2020). Chromatin displays two main types of organization: euchromatin and heterochromatin, the latter showing higher condensation and lower transcriptional activity (Allshire and Madhani, 2018). Heterochromatin can be further subdivided into constitutive and facultative heterochromatin, which play different roles and display specific chromatin marks (Allshire and Madhani, 2018). Constitutive heterochromatin is highly conserved across different cell types and is tightly involved in the maintenance of genome integrity and chromosome segregation (Janssen et al., 2018); notable examples are pericentric and telomeric regions (Saksouk et al., 2015; Lim and Cech, 2021). On the other hand, facultative heterochromatin differs between cell types, and plays a key role in silencing gene expression, thus governing cell fate (Trojer and Reinberg, 2007). One example is the inactive X chromosome, whose inactivation early during mammalian development is vital for gene dosage compensation in female organisms (Galupa and Heard, 2018).

The specific organization of heterochromatin domains can impact the recruitment and activity of DNA repair factors (Caron et al., 2021; Merigliano and Chiolo, 2021). As such, elucidating how heterochromatin domains are affected by DNA damage and in turn regulate the DNA damage response are important avenues of research.

In DNA repair studies, the induction of DNA damage can be achieved through different chemical, physical or enzymatic approaches, including genotoxic drugs, radiations and nucleases (Chatterjee and Walker, 2017). However, few approaches allow the targeting of specific chromatin regions together with the imaging of the DNA damage response (DDR) at such sites.

One targeted enzymatic approach consists of using sequence-specific or RNA-guided endonucleases such as I-SceI, I-PpoI, AsiSI and CRISPR-Cas9 (Vítor et al., 2020), which can be exploited to induce double-strand breaks (DSBs) in different chromatin contexts (Clouaire et al., 2018; Schep et al., 2021). However, imaging of repair factors at the single cell level can be difficult due to the minute number of induced lesions. This issue can be circumvented by inducing breaks in repetitive sequences (Tsouroula et al., 2016; Yilmaz et al., 2021); however, this option is not always possible or desirable. Moreover, break induction by endonucleases requires time for the enzymes to be expressed and located to the region of interest and the sequence itself can undergo cutting multiple times, which limits the temporal resolution of this approach (Liu et al., 2020; Vítor et al., 2020).

Another targeted approach for DNA damage induction relies on laser micro-irradiation, which couples the induction of DNA damage with confocal imaging (Kim et al., 2019). Even though this technique has lower spatial resolution than endonuclease-based approaches, it displays a much higher temporal resolution, and can be used to assess the recruitment of fluorescently-tagged proteins to damage sites by live imaging, immediately after DNA damage induction. Alternatively, the damaged cells can be fixed and subjected to immunofluorescence to visualize the recruitment of endogenous factors with specific antibodies (Kim et al., 2019). Furthermore, laser micro-irradiation allows the targeting of sub-nuclear compartments after their visualization through Hoechst staining or through the expression of fluorescently-tagged proteins that are enriched in the chromatin region of interest. This is particularly helpful for targeting heterochromatin regions, such as those described in this work, namely constitutive pericentric heterochromatin and facultative heterochromatin on the inactive X chromosome.

We assess the DDR in these heterochromatin domains after induction of two types of DNA damage: UVC-induced photolesions and DSBs. Typical photolesions induced by UVC irradiation are cyclobutane pyrimidine dimers (CPDs) and, in a lower proportion, 6-4 photoproducts (6-4PPs) (Dinant et al., 2007). We inflict photolesions by exploiting a UVC laser coupled to a confocal microscope with quartz optics. On the other hand, induction of DSBs can be obtained through different laser micro-irradiation conditions such as UVA, 405 nm or multi-photon approaches using near infrared lasers (Dinant et al., 2007). It should be noted that all of these approaches lead to many types of DNA damage, including DSBs (Dinant et al., 2007). Generally, UVA and 405 nm laser micro-irradiation are carried out after cell sensitization either with the nucleotide analog bromodeoxyuridine (BrdU) or with the DNA minor groove-binding agent Hoechst, because the generation of DSBs without these compounds would require a much higher irradiation dose, and thus increase phototoxicity (Mistrik et al., 2016). The sensitization conditions and the micro-irradiation setup (laser wavelength, power, iterations) determine the type and number of breaks that are inflicted (Dinant et al., 2007).

In this method paper, we take advantage of laser micro-irradiation to induce damage in heterochromatin regions specifically and visualize DSB repair pathways in facultative heterochromatin and the response to photolesion induction in constitutive heterochromatin.

## 2 Materials and Equipment

### 2.1 Cell Culture

1. NIH/3T3 mouse fibroblasts stably expressing a GFP-tagged human DDB2 (DNA Damage Binding protein 2) transgene (Fortuny et al., 2021) to monitor the response to photolesions in constitutive heterochromatin.
2. Female human RPE-1 cells stably expressing GFP-Ku70 [endogenous tagging (Brown et al., 2015), gift from S. P. Jackson] or nucleofected with EGFP-POLQ plasmid [(Zhou et al., 2021), gift from R. Ceccaldi)] 16 h before micro-irradiation to monitor the response to DSBs in facultative heterochromatin. The POLQ gene encodes DNA polymerase theta (Polθ).
3. RPE-1 cells stably expressing MacroH2A1.2-GFP (exogenous expression) were obtained by antibiotic selection of single clones after cell transfection. These cells are used to facilitate the localization of the inactive X chromosome in live imaging and immunofluorescence analyses.
4. Dulbecco’s Modified Eagle Medium with high glucose, GlutaMAX, sodium pyruvate, and phenol red (DMEM high glucose GlutaMAX™ Supplement, Gibco) supplemented with 10% fetal bovine serum (Eurobio Scientific), 100 U/ml penicillin and 100 μg/ml streptomycin (Sigma-Aldrich). Selection antibiotics added for NIH/3T3 GFP-DDB2 cells: 200 μg/ml hygromycin B (Euromedex). Selection antibiotics added for RPE-1 MacroH2A1.2-GFP cells: 400 μg/ml G418 disulfate salt solution (Sigma-Aldrich)
5. Dulbecco’s Phosphate-Buffered Saline without calcium, magnesium, and phenol red (DPBS 1×, Gibco).
6. Humidified incubator (37°C, 5% CO_2_, HeraCell 150).
7. Six-well cell culture plates (TPP), four-well cell culture plates (Nunc).
8. Round glass coverslips 12 mm diameter, thickness No. 1.5 (Thorlabs) for laser micro-irradiation in facultative heterochromatin.
9. Round quartz coverslips 25 mm diameter, thickness No. 1 (01019T-AB, SPI) for UVC laser micro-irradiation in constitutive heterochromatin (see Note a)

### 2.2 Reagents

1. Collagen Type 1 and Fibronectin (Sigma-Aldrich), to increase the adherence of mouse NIH/3T3 cells to coverslips
2. 10 mg/ml Hoechst 33258, Pentahydrate (bis-Benzimide) (Sigma-Aldrich)

### 2.3 Plasmids

1. PiggyBac-eGFP-POLQ-FLAG-P2A-BLAST (Zhou et al., 2021, gift from R. Ceccaldi)
2. MacroH2A1.2-GFP plasmid [Addgene #45169 (Chadwick and Willard, 2001)]

### 2.4 Nucleofection Reagents

1. Neon™ Transfection System 10 µl Kit, Pipette, Pipette Station, Transfection Tubes (ThermoFisher Scientific)
2. Antibiotic-free Dulbecco’s Modified Eagle Medium with high glucose, GlutaMAX, sodium pyruvate, and phenol red (DMEM high glucose GlutaMAX™ Supplement, Gibco) supplemented with 10% fetal bovine serum (Eurobio Scientific)

### 2.5 Equipment

#### 2.5.1 Laser Micro-Irradiation in Facultative Heterochromatin

1. Zeiss LSM780 confocal microscope with a temperature-controlled chamber, CO_2_ supply and Zen software (Carl Zeiss) (see Note b)
2. Laser: 405 nm laser diode (30 mW)
3. Imaging lasers: diode 405 nm (30 mW), Argon 488 nm (25 mW), Argon 561 nm (15 mW)
4. Objective: Plan-Apochromat ×63/1.40 Oil
5. Immersol 518F oil for immersion at 37°C (Carl Zeiss)
6. Ludin chamber type 3 (12 mm diam., Life imaging services)
7. Microscope stage insert for Ludin chamber 160 × 110 mm (Life imaging services)

#### 2.5.2 UVC Laser Micro-Irradiation in Constitutive Heterochromatin

1. Fully motorized Zeiss LSM700 Axio Observer Z1 confocal microscope (Carl Zeiss) adapted for UVC transmission with all-quartz optics, a temperature-controlled chamber, CO_2_ supply and Zen software (Carl Zeiss) (see Note c).
2. UVC laser: 2 mW pulsed (7.8 kHz) diode-pumped solid-state laser emitting at 266 nm (Rapp OptoElectronics, Hamburg GmbH), directly coupled to the microscope stand. A neutral density filter OD1 (10% T) can be added to the light path. The UVC laser is fixed but the position of the damage spot can be precisely controlled by moving the motorized stage of the microscope via a custom macro on Zen software (see Note d).
3. Imaging lasers: diode 405 nm (5 mW), Argon 488 nm (10 mW).
4. Objective: Quartz ×40/0.6 Ultrafluar glycerol objective (Carl Zeiss) (see Note e).
5. Glycerol for immersion.
6. Chamlide CMB 35 mm dish type 1-well magnetic chamber for round coverslip (Live Cell Instrument).
7. SI-K-10 universal stage insert for 35 mm Chamlide chamber (Live Cell Instrument).

### 2.6 Software for Image Processing and Analysis

1. ImageJ/FiJi software (United States National Institutes of Health, Bethesda, Maryland, United States, http://imagej.nih.gov/ij/).
2. GraphPad Prism
3. Adobe Photoshop or equivalent image processing software

## 3 Step-by-Step Protocol

### 3.1 Imaging the Response to DNA Double-Strand Breaks in Facultative Heterochromatin

**FIGURE 1:**
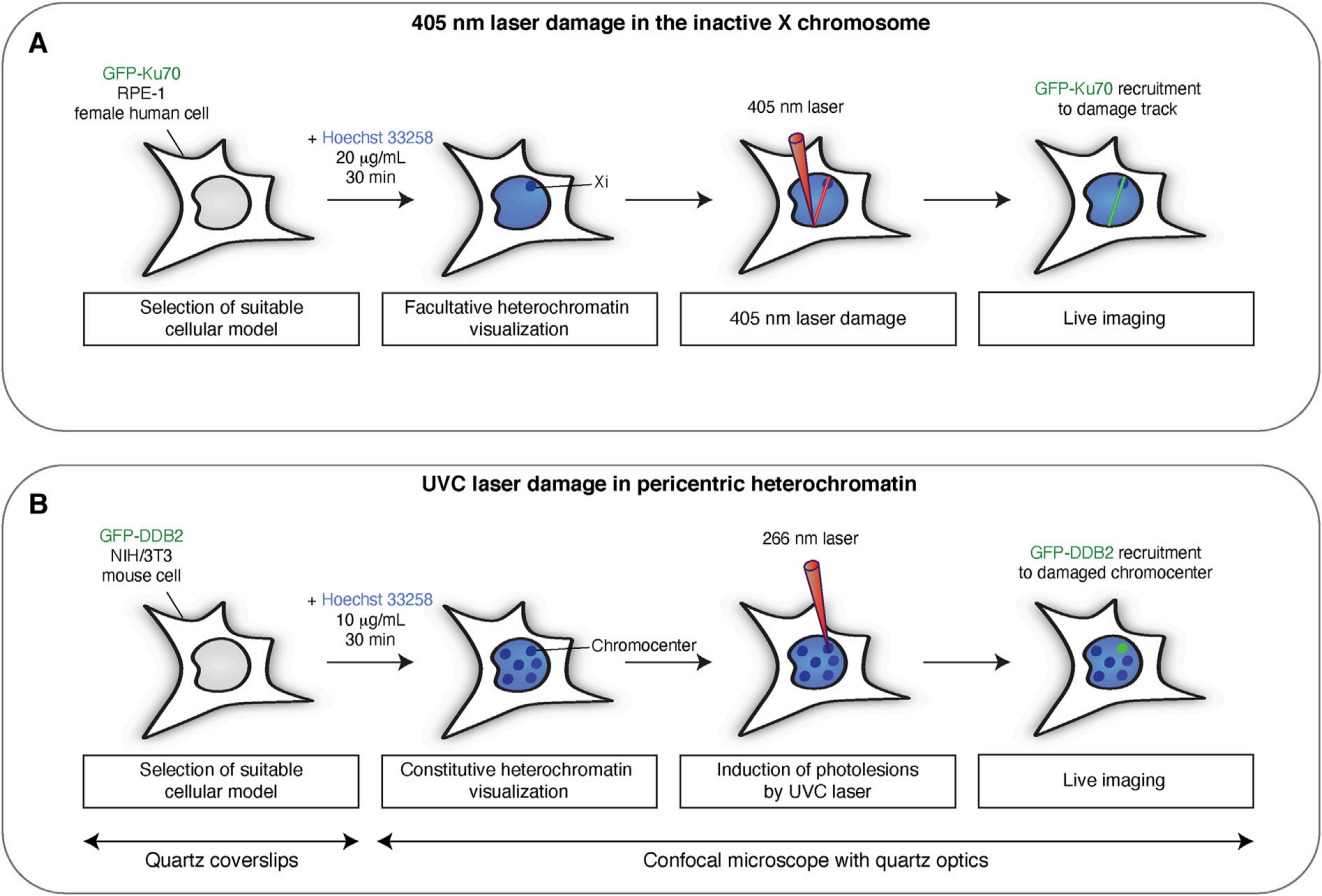
Targeting DNA damage to heterochromatin domains in mammalian cells with laser micro-irradiation. Scheme illustrating the main steps for inducing DNA damage in the inactive X-chromosome (Xi) by 405 nm laser micro-irradiation **(A)** and in pericentric heterochromatin by UVC laser micro-irradiation **(B)**. UVC micro-irradiation requires quartz coverslips and quartz optics since UVC light does not penetrate conventional glass materials.

**FIGURE 2:**
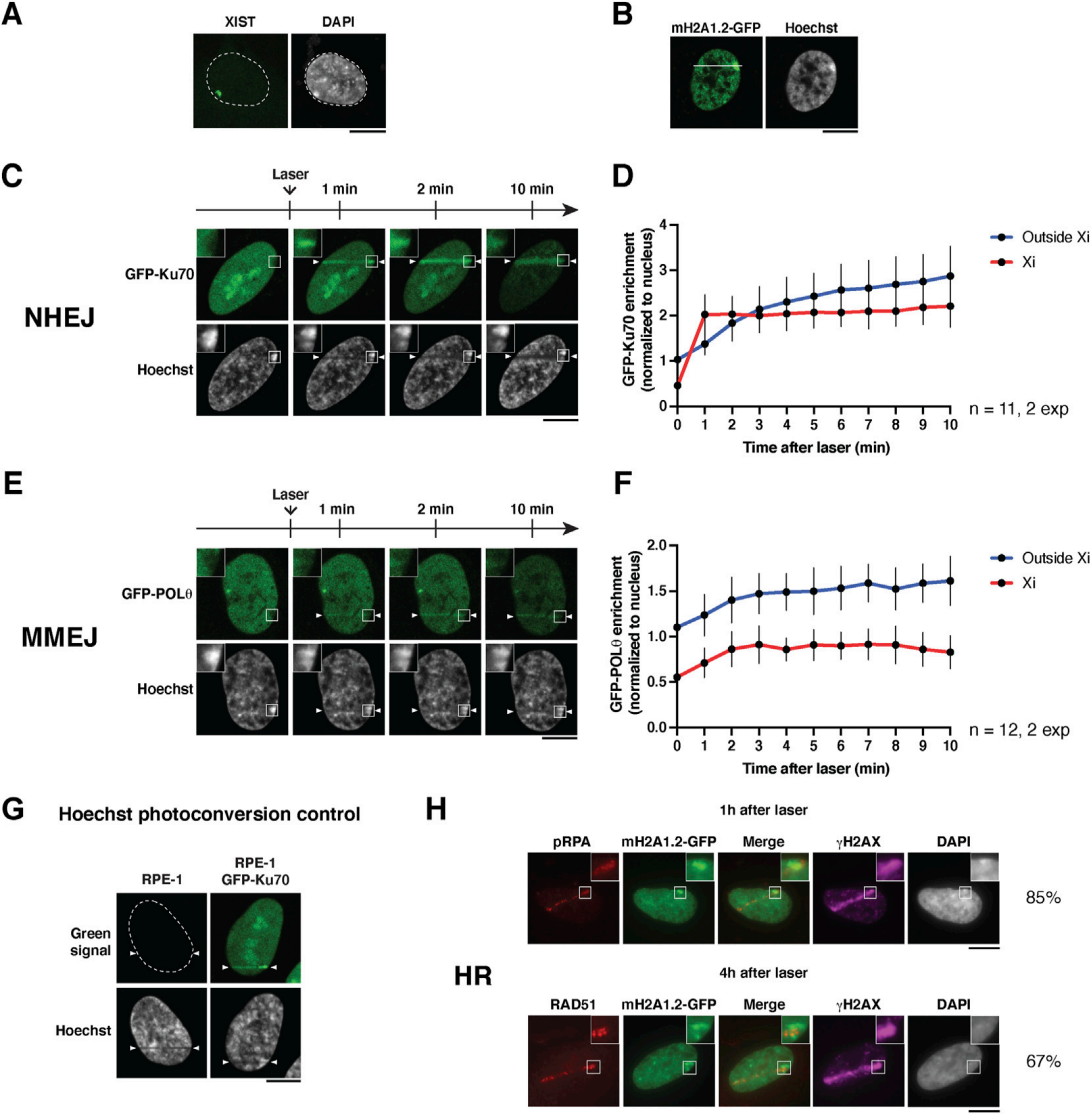
Imaging the recruitment of DSB repair factors to the inactive X chromosome. **(A)** RNA-FISH staining for XIST RNA on the inactive X chromosome in RPE-1 cells using the method described in Chaumeil et al. (2008). The XIST cloud colocalizes with the most intense DAPI region in the nucleus. The microscopy image is a maximum intensity projection of 25 z-planes. **(B)** Nucleus of an RPE-1 cell stably expressing macroH2A1.2-GFP and stained with Hoechst. A white line depicts how to position the laser line to damage the Xi. **(C,E)** Live cell imaging showing the recruitment of the indicated GFP-tagged NHEJ and MMEJ factors to the inactive X chromosome (Xi) following 405 nm laser micro-irradiation in human RPE-1 cells. **(D,F)** Quantifications of the signals in the Xi (red) and outside the Xi (blue) as a function of time after laser damage are shown for each repair factor. Data are presented as mean values ± SD from a total of n nuclei scored in two independent experiments. **(G)** Hoechst photoconversion control after 405 nm micro-irradiation in RPE-1 cells (parental untransfected cell line vs cells expressing GFP-Ku70). Imaging settings were kept the same for both cell types. **(H)** Recruitment of the indicated HR factors in the inactive X chromosome (Xi) analyzed by immunofluorescence, 1 h (pRPA) or 4 h (RAD51) after 405 nm laser micro-irradiation in RPE-1 cells stably expressing GFP-tagged macroH2A1.2 (mH2A1.2-GFP). The percentages indicate the proportion of HR-prone cells (S/G2) that display recruitment of the respective HR factor to the damaged Xi. Antibodies used: primary antibodies anti-phospho-RPA32 Ser4/8 (Bethyl Laboratories A300-245A, 1:1,000), anti-RAD51 (Abcam ab176458, 1:1,000), anti-γH2A.X (Merck Millipore 05-636, 1:1,000); secondary antibodies anti-rabbit Alexa Fluor 568 (Invitrogen A11036, 1:1,000), anti-mouse Alexa Fluor 647 (Invitrogen A21236, 1:1,000). The position of the laser track is indicated by arrowheads. All microscopy images are confocal sections unless stated otherwise. Scale bars, 10 μm.

Female human cells possess two X chromosomes, one of which is inactivated to form a facultative heterochromatin domain termed the inactive X chromosome (Xi). We use female human RPE-1 cells and induce DSBs in the Xi by laser micro-irradiation on a confocal microscope (Figure 1A). We then follow the recruitment of different DSB repair factors by live imaging or by immunofluorescence. The Xi can be identified as a DNA-dense region in the nucleus, which is coated by the X inactive specific transcript (XIST RNA, Figure 2A).

1. Seed cells on glass coverslips at least 24 h before the experiment so that they reach 70–80% confluency at the time of laser micro-irradiation
2. On Zen software, set the parameters for live imaging and laser micro-irradiation:
  - Objective: Plan-Apochromat ×63/1.40 Oil
  - Channels: Hoechst, GFP
  - Imaging parameters: 12 bits, 1,024 × 1,024 pixels, Zoom 2X, scan time 5.03 μs, 1.02 μs/pixel dwell time
  - Bleaching parameters: 405 nm laser, 13.6 μs/pixel dwell time, two iterations at 20% laser power for homologous recombination factors (RPA, RAD51), 20 iterations at 25% laser power for end-joining factors (Ku70, Polθ), the accumulation of which is more difficult to detect. Start bleaching after 1 picture (see Note f).
  - Time series: 1 picture/min for 10 min for live imaging of end-joining factors (see Note g). For homologous recombination (HR) factors, take two pictures in a row, one before and one after micro-irradiation, with no interval between the two images.
  - Activate the autofocus mode to keep the cells in focus throughout the experiment
3. Incubate one coverslip with Hoechst 33258 (20 μg/ml final in DMEM) at 37°C, 5% CO_2_ for 30 min (see Note h). Hoechst sensitizes cells to laser damage by binding to the DNA minor groove.
4. Remove Hoechst-containing medium, wash coverslip with DPBS 1X
5. Transfer the coverslip to the Ludin chamber with 1 ml of fresh DMEM and place the Ludin chamber on the microscope stage with controlled temperature and CO_2_ conditions (37°C, 5% CO_2_).
6. Locate a nucleus of interest with a clear Xi (Hoechst-dense region or macroH2A1.2-GFP-enriched focus).
7. Select the area to be bleached with a rectangular selection of 1 pixel in height (roughly 0.13 μm). The line should be drawn to match the direction of laser scanning. In addition, the line should cut across the Xi and a significant portion of the nucleus to be able to compare repair factor accumulation in and outside the Xi (Figure 2B).
8a. For end-joining factors, start the experiment on Zen software to initiate the bleaching (laser damage) followed by acquisition of images in time series (1 image/min for 10 min) and then repeat steps 6–8 to reach a total of twenty damaged nuclei (see Note i). Acquisitions are performed in 2D at the best focal plane. Continue to point 9a for analyses of live imaging pictures.
8b. For HR factors, up to twenty nuclei of interest are micro-irradiated. The coverslip is then transferred to a 4-well plate containing the appropriate culture medium and left in the incubator at 37°C and 5% CO_2_ for 1–4 h recovery post laser damage, HR being a slower process than end-joining. Continue to point 9b for immunofluorescence.
9a. Image analysis is carried out with FiJi software: on each image file, draw circular regions of interest of 16 pixels in diameter in the background (region distant from other nuclei), in the Xi, and in the laser track outside the Xi. Measure mean fluorescence intensities in each region of interest and in the entire nucleus, in all channels. This step is repeated for every timepoint and for 10–20 cells from at least two independent experiments. All cells are damaged independently of each other, as such they can be considered as independent events. For this reason, statistical tests are carried out on the means of all damaged cells instead of the means of independent experiments. Furthermore, cell-to-cell variation appears higher than between experiments.
To assess the local enrichment of repair factors on the laser track, the mean fluorescence intensities in the damaged Xi region and in the damaged track outside the Xi are normalized to the mean fluorescence intensity measured in the entire nucleus, after background fluorescence subtraction. This calculation allows merging the data from different nuclei by circumventing potential variations in fluorescence intensities between nuclei. Results are then plotted as a function of time after micro-irradiation. This allows the averaging of values coming from different image files in the same graph. Statistical analyses are run with GraphPad Prism. Selected images are processed and mounted with Adobe Photoshop.
9b. Complementary to live cell imaging, the cells can be fixed with 2% paraformaldehyde at different time points after laser micro-irradiation and processed for immunofluorescence to detect DSB repair factors on laser tracks. Co-detection of the DNA damage marker γH2A.X can be used to visualize laser tracks, except at late time points post laser micro-irradiation at high laser damage (end-joining settings) because γH2A.X becomes pan-nuclear. Localization of the Xi through DAPI staining can be challenging after laser micro-irradiation and immunofluorescence. For this reason, we employ RPE-1 cells stably expressing MacroH2A1.2-GFP, a histone variant that is highly enriched in the Xi (Costanzi and Pehrson, 1998).

The time required to go through steps 6 to 8a is around 10 min per nucleus, and 3–4 h to image 20 damaged nuclei. Note that finding nuclei to be damaged is more challenging in transiently transfected cells due to the high variability in expression between nuclei. If immunofluorescence is performed after laser damage instead of live imaging, going through steps 6–8b requires around 20 s per nucleus, and 15 min to damage 20 nuclei.

### 3.2 Imaging the Response to Photolesions in Constitutive Heterochromatin

Mouse fibroblasts exhibit clusters of pericentric heterochromatin, called chromocenters, that are easily detectable by Hoechst or DAPI staining. However, mouse fibroblasts express the UV damage sensor protein DDB2 at very low levels and ectopic expression of DDB2 is thus necessary to restore full proficiency of the nucleotide excision repair pathway (Fortuny et al., 2021). Here, we describe how to generate photolesions in pericentric heterochromatin by targeting a UVC laser to chromocenters (Figure 1B).

1. Coat quartz coverslips (see Notes a, j) with 20 μg/ml Collagen Type 1 and 2 μg/ml fibronectin (Sigma-Aldrich) in DPBS 1× for at least 1 h at 37°C to increase adherence of mouse cells.
2. Seed NIH/3T3 GFP-DDB2 cells on coated quartz coverslips at least 24 h before the experiment so that they reach 70–80% confluency at the time of micro-irradiation.
3. On Zen software, set up the following parameters for live imaging and UVC micro-irradiation:
  - Objective: ×40/0.6 Ultrafluar glycerol with quartz lenses (see Note e)
  - Two channels: Hoechst and GFP
  - Imaging parameters: 8 or 12 bits, 1,024 × 512 pixels, Zoom 4X, 12.61 μs/pixel dwell time
4. Incubate cells on coverslips with Hoechst 33258 (10 μg/ml final) in DMEM at 37°C, 5% CO_2_ for 30 min.
5. Transfer one coverslip to the Chamlide magnetic chamber with 1 ml Hoechst-containing DMEM and place the chamber on the microscope stage insert with controlled temperature and CO_2_ conditions (37°C, 5% CO_2_).
6. Locate the cell nuclei based on Hoechst staining, visualize the chromocenters (Hoechst-dense regions) and select a nucleus of interest.
7. Acquire a picture of the nucleus before damage using Hoechst and GFP channels.
8. Turn on the UVC laser, insert the neutral density filter in the light path, and close the lamp shutter (see Note k).
9. On the UV macro, mark the chromocenter to be damaged, and irradiate with the UVC laser for 50 ms (see Notes l, m). Several nuclei can be damaged in the same field.
10. Acquire a picture immediately after the damage using Hoechst and GFP channels (30 s time point) to verify that the damage colocalizes with the targeted chromocenter.
11. Acquire post damage pictures in time series using Hoechst and GFP channels (1 image/3 min for 30 min) (see Note n) for up to 10 nuclei. Acquisitions are performed in 2D at the best focal plane. The focus can be re-adjusted manually at each time point if the autofocus mode is not activated.
12. Image analysis is performed with FiJi software. To measure GFP-DDB2 intensity at UV spots, select the damaged area with the magic wand based on the GFP-DDB2 signal and measure GFP total fluorescence intensity (integrated density) in this region of interest. This step is repeated for every time point and for up to 10 cells per experiment. For the time point before laser micro-irradiation, the region of interest is the same as the one used for the 30 s image. The fluorescence intensities normalized to before damage are plotted as a function of time after micro-irradiation. To measure the area of the damaged chromocenter, select the damaged chromocenter with the magic wand based on the Hoechst signal and measure the area at each time point. The areas normalized to before damage are plotted as a function of time after micro-irradiation. Selected images are processed and mounted using Adobe Photoshop. As for 405 nm laser micro-irradiation, each cell is damaged independently. Therefore, they can be considered as independent events. For this reason, statistical tests are carried out on the means of all damaged cells instead of the means of independent experiments.

The time required to go from step 6 to 10 is around 3 min per nucleus, and it takes about 5 h to image 10 damaged nuclei (step 11). It is not advisable to perform longer experiments because the viability of the cells can be negatively impacted by the Hoechst present in the medium.

Complementary to live cell imaging, the cells can be fixed with 2% paraformaldehyde at different time points after UVC micro-irradiation and processed for immunofluorescence to detect UV damage repair factors on damaged chromocenters.

## 4 Anticipated Results

### 4.1 Recruitment of DSB Repair Factors to the Inactive X Chromosome After Laser Micro-Irradiation

Using the method described above, we followed the recruitment of DSB repair factors to the damaged Xi in real-time in RPE-1 cells expressing fluorescently tagged repair factors, Ku70 and Polθ (Figures 2C,E). These factors belong to two distinct DSB repair pathways, non-homologous end joining (NHEJ), and microhomology-mediated end joining (MMEJ), respectively (Scully et al., 2019).

We observed that both factors were excluded from the Xi in non-irradiated cells, likely due to the high compaction state of this chromatin compartment. Within minutes after micro-irradiation, we observed the recruitment of Ku70 and Polθ to laser tracks. Interestingly, both DSB repair factors showed an accumulation on the damaged Xi, with similar kinetics to non-Xi chromatin for Polθ and even faster kinetics for Ku70 (Figures 2D,F). This is an intriguing observation which could indicate that the chromatin context of the Xi allows faster Ku70 accumulation in response to DSBs.

From a technical point of view, we verified that the increase of green fluorescent signal on the laser track was not due to Hoechst photoconversion (Figure 2G), a phenomenon in which UV irradiation can induce a chemical change in the Hoechst molecule that leads to emission of green and red wavelengths (Hurst and Gasser, 2019).

We also attempted to follow by live imaging the recruitment of another repair factor, RAD51, involved in DSB repair by homologous recombination (HR). However, even when transfecting cells with a plasmid encoding GFP-RAD51 under the control of a weak promoter (Foertsch et al., 2019), the levels of expression were still too high and led to the formation of RAD51 filaments even in undamaged cells, which hampered the study of RAD51 relocalization after laser micro-irradiation. Instead, we assessed the recruitment of two endogenous HR repair factors, phosphorylated RPA (pRPA32 Ser4/8) and RAD51, by immunofluorescence in RPE-1 cells stably expressing macroH2A1.2-GFP (Figure 2H). We observed the recruitment of pRPA and RAD51 to laser tracks 1 and 4 h after laser micro-irradiation, respectively, in a subset of cells, HR being restricted to S/G2 cell cycle stages (Scully et al., 2019). Among cells that displayed pRPA and RAD51 recruitment on laser tracks, a vast majority showed HR factor recruitment to the damaged Xi. Notably, RAD51 recruitment coincides with a region of the Xi showing lower macroH2A1.2 levels, reflecting either a decompaction of facultative heterochromatin or a local depletion of this facultative heterochromatin mark during the late steps of HR repair.

Together, these results indicate that the three DSB repair pathways examined - NHEJ, MMEJ and HR - operate efficiently in facultative heterochromatin domains. To strengthen these findings, it will be important to monitor the recruitment of other repair factors acting in the same pathways, through real-time imaging on live cells or immunofluorescence on fixed cells. It will also be interesting to evaluate the relative contribution of each of these repair pathways to the maintenance of genome and epigenome integrity in facultative heterochromatin domains.

### 4.2 Decompaction of Constitutive Heterochromatin and Recruitment of Repair Factors Following UVC Laser Micro-Irradiation

**FIGURE 3:**
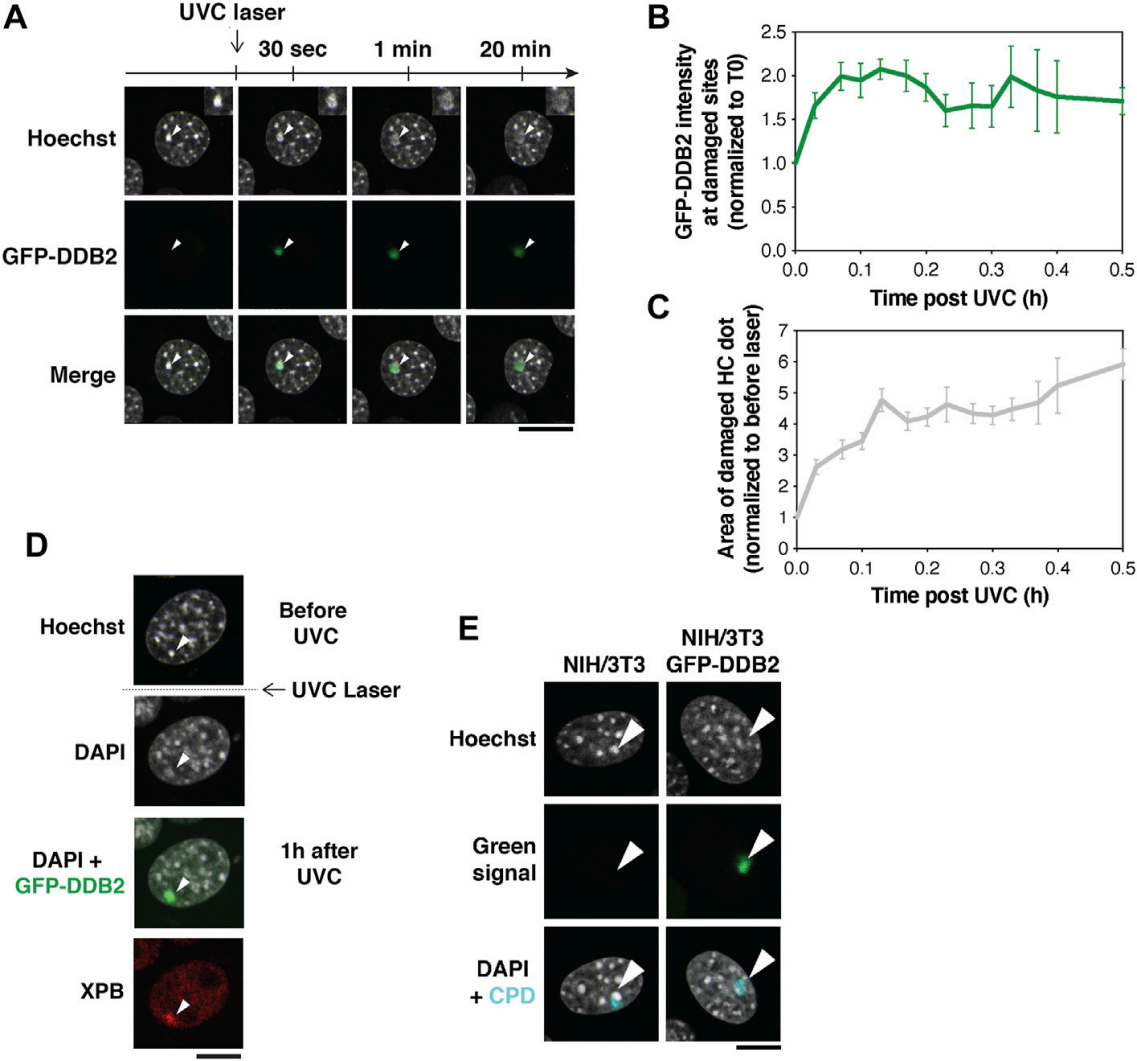
Pericentric heterochromatin decompaction and recruitment of UV damage repair proteins. **(A)** Decompaction of pericentric heterochromatin following UVC laser damage analyzed in NIH/3T3 cells expressing GFP-DDB2 (UV damage sensor). **(B,C)** Quantification of GFP-DDB2 intensity in damaged heterochromatin **(B)** and chromocenter area **(C)** relative to before damage. Data are presented as mean values ± SEM from 12–45 cells (depending on the timepoint) scored in at least eleven independent experiments. **(D)** Recruitment of XPB repair protein analyzed by immunofluorescence 1 h after UVC laser micro-irradiation in NIH/3T3 GFP-DDB2 cells. Antibodies used: primary antibody anti-XPB (Santa cruz Biotechnology sc-293, 1:400), secondary antibody anti-rabbit Alexa Fluor 594 (Invitrogen A11037, 1:1,1000). **(E)** Hoechst photoconversion control after UVC laser micro-irradiation in NIH/3T3 cells (parental cell line vs cells expressing GFP-DDB2) followed by immunofluorescence for UV lesions (Cyclobutane Pyrimidine Dimers, CPD) 30 min after irradiation. Antibodies used: primary antibody anti-CPD (Cosmo Bio CAC-NM-DND-001, clone TDM2, 1:1,000), secondary antibody anti-mouse Alexa Fluor 647 (Invitrogen A21236, 1:1,000). Imaging settings were kept the same for both cell types. The arrowheads point to damaged chromocenters. All microscopy images are confocal sections. Scale bars, 10 μm. Data from Fortuny et al. (2021).

By targeting UVC laser damage to pericentric heterochromatin domains in NIH/3T3 GFP-DDB2 cells, we observed a rapid recruitment of the UV damage sensor DDB2 to the damaged chromocenters, detectable within seconds (Figures 3A,B). This was followed by a marked decompaction of the damaged chromocenters as revealed by a 6-fold increase in their area during the first 30 min post irradiation (Figure 3C).

The decompaction of heterochromatin was shown to be triggered by DDB2 and likely facilitates the recruitment of downstream repair factors to damaged chromocenters (Fortuny et al., 2021). This is exemplified by the accumulation of the repair helicase XPB, detected by immunostaining on the damaged chromocenter 1 h after UVC laser damage (Figure 3D).

While 405 nm laser micro-irradiation on Hoechst-stained cells generates DSBs, DSBs are not detectable in response to the UVC laser (Fortuny et al., 2021). Moreover, similar to what observed with the 405 nm laser, no photoconversion of Hoechst by UVC light was detected (Figure 3E) (Fortuny et al., 2021).

These results demonstrate that UV damage repair factors can access the core of pericentric heterochromatin domains and illustrate the power of UVC laser micro-irradiation to probe dynamic changes in constitutive heterochromatin organization following DNA damage.

## 5 Notes

a. UVC light does not go through glass. Quartz coverslips are used to allow the transmission of UVC light through the coverslip to the sample. They can be recycled and re-used after UVC damage (if samples were not fixed for immunofluorescence). For this, the coverslips should be rinsed for 10 min in 1% Sodium Dodecyl Sulfate, then three times for 10 min in H_2_O and twice for 10 min in 100% ethanol. After drying, the coverslips are autoclaved and can be re-used in the next experiment.
b. Any equivalent confocal microscope can be used as long as it is equipped with a suitable laser (355–405 nm).
c. Any equivalent confocal microscope can be used as long as it can be coupled to a 266 nm laser and its optics are changed to quartz. Local UVC irradiation can also be performed at the bench with a UVC lamp (254 nm) through micropore filters (Moné et al., 2001).
d. A UGA point scanner device (Rapp OptoElectronic) can be implemented to control the position of the UVC laser.
e. A ×100/1.2 glycerol objective with quartz lenses can also be used to achieve better resolution.
f. The laser power, iterations and speed will likely be different in other experimental setups. To identify the optimal bleaching parameters, the experimenter should use cells transiently or stably expressing fluorescently-tagged repair factors and test different parameters. To minimize phototoxicity, one should use the lowest parameters that lead to detectable and time-resolved recruitment of repair factors. Fluorescently-tagged NBS1 can be used in the first place to adjust laser parameters because NBS1 binds broken DNA ends directly, is recruited quickly and NBS1 accumulation at DSBs is easy to detect. Then fluorescently-labelled Ku70 can be used to fine-tune laser settings for end-joining factors, RPA for HR factors.
g. While laser micro-irradiation with our settings does not lead to cell death, repeated imaging cycles can lead to cell mortality due to phototoxicity. For this reason, the laser power, number and duration of acquisitions should be minimized. For fast recruiting proteins like end-joining factors, the interval between pictures can be decreased or even set to zero to take pictures as fast as possible and obtain higher temporal resolution. Additionally, the speed of acquisition can be increased, at the expense of image resolution.
h. The nucleotide analog bromodeoxyuridine (BrdU) can be used instead of Hoechst to sensitize cells. In this case, BrdU should be added to the cell culture medium 24 h before the experiment at a final concentration of 10 μM. Unlike Hoechst, BrdU cannot be visualized at the microscope; therefore, a cell line expressing fluorescently tagged Xi markers can be used to identify the Xi. The parameters for irradiation are also different, due to the different type of sensitization. Generally, a higher power and increased number of iterations (e.g., 40% power, 50 iterations) are needed for BrdU.
i. A total of 10 damaged cells is enough to have good assessment of repair factor recruitment to laser tracks (Kim et al., 2019), so damaging 20 cells is a good trade-off between experimental duration and statistical power.
j. Coverslips can be marked with a diamond pen before seeding the cells to facilitate the localization of damaged cells if immunofluorescence is performed after laser damage.
k. For safety reasons, it is important to close the microscope chamber during the UVC micro-irradiation procedure and the lamp shutter should also be closed to avoid transmission of the UVC light through the eyepieces.
l. The duration of UVC laser exposure may be adjusted if using a different microscope set up. To identify the optimal conditions, the experimenter should use cells transiently or stably expressing fluorescently-tagged UV damage sensors like GFP-DDB2 or GFP-XPC and test different exposure times to the UVC laser. To minimize phototoxicity, one should use the lowest exposure time that leads to detectable recruitment of repair factors.
m. The damage spot is 2 μm in diameter corresponding to ca. 2% of the nuclear volume. The UVC dose delivered at the site of laser micro-irradiation is estimated at 600 J/m^2^ and does not cause major cytotoxicity in the time frame of the analysis.
n. For longer time courses, you can save the position of the damaged nucleus before moving to the next field. Repeat steps 7–10 for up to ten nuclei. The time series can be extended to several hours/overnight by spacing out the acquisitions to avoid photobleaching.

## 6 Conclusion

The laser micro-irradiation-based approaches presented here are powerful methods to induce targeted DNA damage to specific nuclear compartments such as heterochromatin domains and to study the spatio-temporal dynamics of DNA repair factor recruitment to these domains by live imaging. The same procedure can be applied to any other protein that accumulates to or is evicted from irradiated sites, and to monitor the dynamics of histone variants and chromatin marks over time. For a deeper understanding of the mechanisms underlying heterochromatin repair, this protocol can be combined with siRNA-mediated depletion or chemical inhibition of repair factors and histone modifying enzymes (Fortuny et al., 2021).

The main advantage of laser micro-irradiation is that it allows the targeting of nuclear sub-compartments of interest and the tracking of the repair response in real-time in single cells, thus circumventing the confounding effects of heterogeneous responses in cell populations. In addition, 405 nm laser micro-irradiation is quite straightforward to implement since it does not require exclusive materials and equipment, and can be applied to virtually any confocal microscope. UVC laser micro-irradiation, however, requires a UVC laser and a confocal microscope adapted with quartz optics. A limitation of these microscopy-based approaches is that they do not offer sufficient resolution for analyzing repair responses at the nucleosomal level. For studying heterochromatin repair at increased resolution, complementary approaches using sequence-specific DSB induction by nucleases can be employed, combined with the mapping of chromatin-bound proteins.

## Data Availability

The original contributions presented in the study are included in the article, further inquiries can be directed to the corresponding author.
